# Joint effects of nulliparity and other breast cancer risk factors

**DOI:** 10.1038/bjc.2011.286

**Published:** 2011-08-02

**Authors:** S Opdahl, M D K Alsaker, I Janszky, P R Romundstad, L J Vatten

**Affiliations:** 1 Department of Public Health and Community Medicine, Faculty of Medicine, Norwegian University of Science and Technology, MTFS, Trondheim N-7491, Norway; 2 Department of Oncology, St. Olavs Hospital, Trondheim N-7006, Norway; 3 Department of Public Health Sciences, Karolinska Institutet, Stockholm SE-171 77, Sweden

**Keywords:** breast cancer risk, nulliparity, synergism, attributable proportion, epidemiology

## Abstract

**Background:**

Pregnancy may reduce breast cancer risk through induction of persistent changes of the mammary gland that make the breast less susceptible to carcinogenic factors. It is not known to what extent the effects of parity are independent of other breast cancer risk factors.

**Methods:**

In a Norwegian cohort of 58 191 women (2890 breast cancers), we assessed whether the effects of parity on postmenopausal breast cancer risk may be modified by menstrual and anthropometric factors. We calculated attributable proportions due to interaction as a measure of synergism.

**Results:**

Parity, height, body mass index (BMI), age at menarche and menopause were all associated with breast cancer risk in the expected directions. For BMI, follow-up was stratified into two age groups because of non-proportional hazards. We found that nulliparity and overweight may amplify each other's effect on breast cancer risk among women after 70 years of age (attributable proportion 0.21, 95% confidence interval 0.04–0.39). There was some indication that parity and age at menopause may antagonise each other's effect. Effects of parity were largely unaffected by age at menarche and height.

**Conclusion:**

Nulliparity and overweight may have a synergistic effect on breast cancer risk in elderly women. If confirmed by others, the findings may help disentangle the interplay of different causes of breast cancer.

The protective effect of pregnancies against breast cancer was first described as early as 1926 (Press and Pharoah, 2010), and was confirmed through a large number of subsequent studies (Kelsey *et al*, 1993). The underlying mechanisms may include differentiation of the mammary epithelial cells, reduced number of mammary stem cells, altered mammary response to oestrogen, and reduced levels of circulating hormones (Britt *et al*, 2007).

If the breast undergoes permanent changes during pregnancy, it is possible that the mammary gland's susceptibility to other important exposures could also be altered by pregnancy-related factors (Britt *et al*, 2007). However, it is unknown whether effects of parity can be modified by other risk factors for breast cancer. If such interactions were identified, our understanding of possible underlying mechanisms may be advanced, which could eventually lead to better preventive strategies.

Relatively few studies have simultaneously assessed the separate and joint effects of parity and other established risk factors, including age at menarche and menopause (La Veccia *et al*, 1992; Hirose *et al*, 2003), and body mass index (BMI) and height (Van den Brandt *et al*, 2000; Hirose *et al*, 2003). Only one of the studies was based on prospectively collected data (Van den Brandt *et al*, 2000), and the testing of heterogeneity of relative risks that was performed in previous studies may be of limited use in assessing causal inference related to interactions (Ahlbom and Alfredsson, 2005; Greenland *et al*, 2008).

Therefore, we have studied whether effects of parity on breast cancer risk may be modified by menstrual or anthropometric factors in a long-term follow-up of a large historical cohort.

## Materials and methods

### Study population and follow-up

The study population consists of women from three Norwegian counties (Nord-Trøndelag, Vestfold, and Aust-Agder) who were born between 1886 and 1928. They were invited to a breast cancer screening organised by the Norwegian Cancer Society between 1956 and 1959. The study has been described in detail elsewhere (Kvåle *et al*, 1987). Briefly, the participants were offered a clinical examination by a medical doctor, and information on reproductive history and demographic factors was collected during structured interviews, conducted by trained personnel. In the interview, information was recorded on age at menarche, number of full-term pregnancies, age at first full-term pregnancy, previous surgery or disease of the breasts or genital organs, age at menopause, marital status, place of residence, and the participant's own or her husband's occupation.

Information on anthropometric factors was added to the database following a compulsory mass examination for tuberculosis that was conducted between 1963 and 1975. That examination included height measured to the nearest centimetre and weight measured to the nearest kilogram on regularly calibrated scales (Tretli, 1989).

At the population census in 1960, a unique 11-digit identity number was assigned to all Norwegian citizens. We used the identity number to link study participants to information on breast cancer incidence at the Cancer Registry of Norway, which also includes information on vital status and emigration, as recorded by the Population Registry at Statistics Norway. Invasive breast cancer was registered according to the International Classification of Diseases, 7th edition (code 170). Among 84 981 invited women who were alive at the census in 1960, 63 041 (74.2%) women had participated at the screening. Among 50 061 (79.4%) of the participating women, anthropometric information was also available.

We started breast cancer follow-up from the age of 55 years. After exclusion of women who died or emigrated before 55 years of age, 62 108 women were eligible for follow-up. Participants were followed from 1 January 1961, or from the age of 55 years, and in analyses that included anthropometric measures, women were followed from the year after the height and weight measurements, or from 55 years of age. End of follow-up was set to the date of breast cancer diagnosis, emigration, death, or to the end of follow-up on 31 December 2008, whichever occurred first.

Among eligible women, we excluded 759 women with a previous breast cancer diagnosis and 3158 women with missing information on the main study factors. A total of 58 191 women were therefore followed up for breast cancer occurrence. Among these women, 20 748 were postmenopausal at the time of the interview and had reported age at menopause. A total of 451 women who reported a surgical menopause (bilateral oophorectomy and/or hysterectomy) were excluded from the analyses related to age at menopause. Thus, the analyses of age at menopause were restricted to 20 297 women with a natural menopause. Information on anthropometric factors was not available for all participants, and among 49 624 eligible women with height and weight measurements, we similarly excluded women with prevalent breast cancer, women with missing information on parity or potentially confounding factors, and women with unreliable height measurements (wearing shoes, abnormal posture). Analyses that included anthropometric measures were therefore based on follow-up of 44 952 women. The number of exclusions and the number of women included in the analyses are summarised in Table 1.

### Parity and other risk factors

We categorised parity as nulliparous (no full-term pregnancy) or parous (one or more full-term pregnancies). In analyses of trend, age at menarche was categorised as ⩽13, 14, 15, ⩾16 years, age at natural menopause as <45, 45–49, 50–54, ⩾55 years, height as <155, 155–159, 160–164, 165–169, ⩾170 cm, and BMI as <25, 25–29, ⩾30 kg m^−2^. When evaluating synergism, menstrual and anthropometric exposures were dichotomised at the median value (age at menarche <15 or ⩾15 years, age at menopause <50 or ⩾50 years, height <161 or ⩾161 cm and BMI <26 or ⩾26 kg m^−2^). Among parous women, number of full-term pregnancies was categorised as 1, 2, 3, ⩾4 and age at first full-term pregnancy as <20, 20–24, 25–29, 30–34, ⩾35 years.

### Statistical analyses

We used Cox proportional hazards models to compute hazard ratios (HRs) with 95% confidence intervals (CIs). The associations of menstrual and anthropometric factors with breast cancer risk were assessed both overall and within the two strata of parity. For each exposure, the category with the largest number of person years was chosen as the reference category. The *P*-values for trend across exposure categories were calculated by treating the categories as a continuous variable. We assessed heterogeneity of the HRs across strata of parity by likelihood ratio tests by comparing models with and without product terms between parity and each exposure variable (where exposure categories were treated as a continuous variable).

Statistical interaction in multiplicative models has no direct causal interpretation, whereas departure from additivity of risks may imply causal interaction (Ahlbom and Alfredsson, 2005; Greenland *et al*, 2008). Therefore, we calculated the attributable proportion due to interaction (API) with 95% CIs to evaluate whether effects of parity could be modified by menstrual or anthropometric factors (Hosmer and Lemeshow, 1992; Andersson *et al*, 2005). The API allows the assessment of effect modification on an additive scale using multiplicative models. An API with a value greater than 0 implies synergism, that is, the effect of the combined exposure is greater than expected from their separate effects, whereas a value below 0 indicates antagonism, that is, less influence by the joint exposure than expected from their separate effects (Andersson *et al*, 2005).

In all analyses, age was controlled for by using it as the time scale in the regression models. In addition, we adjusted for birth cohort (in 5-year categories), marital status (ever or never), county, urban or rural place of residence, and the participant's own or her husband's occupation (professional/private enterprise, manual work, domestic and other work). Age at menarche was considered a potentially confounding factor in analyses of age at natural menopause, height, and BMI, and these analyses were therefore also adjusted for age at menarche. In a separate analysis restricted to parous women, we controlled for potential confounding by number of full-term pregnancies and age at first full-term pregnancy, in the trend analyses of age at menarche, age at natural menopause, height and BMI.

Proportionality between hazards was checked by comparing log minus log plots of survival and by performing tests based on Schoenfeld residuals. Assumptions were met for all exposures except BMI, for which the log minus log survival curves were non-parallel from 70 years and onwards. Others have also found that the positive association of BMI among postmenopausal women may be more pronounced among older women (Yong *et al*, 1996; Galanis *et al*, 1998). On the basis of these previous observations and on the observed non-proportionality, analyses of BMI were stratified by follow-up time into two periods, one from 55–69 years of age, and the other with follow-up from 70 years of age. Each woman contributed person-years to one or both periods, depending on her age at the start and end of follow-up. After stratification, the proportional hazards assumption was met in both age groups.

All analyses were conducted using Stata version 11 for Windows (Stata Corp., College Station, Texas).

### Ethical considerations

The study was approved by the regional committee for medical research ethics and by the Norwegian Data Inspectorate.

## Results

A total of 58 191 women without breast cancer at baseline were followed for 1 400 436 person years with a mean follow-up of 24.1 years (standard deviation, s.d.: 9.7). During follow-up, 2890 women were diagnosed with invasive breast cancer. Mean age at diagnosis was 72.9 years (s.d. 9.4), and overall, 48 945 (84.1%) women died during follow-up. Characteristics of the participants at baseline are shown in Table 2. Compared with parous women, nulliparous women were older and had a lower BMI. They were also more likely to be unmarried and live in an urban community, and less likely to be employed in manual work (the participant's own or her husband's occupation).

The HRs for menstrual and anthropometric risk factors stratified by parity are presented in Table 3. Nulliparous women were at higher risk for breast cancer than parous women. For nulliparous and parous women combined, breast cancer risk was negatively associated with age at menarche, and positively associated with height, age at natural menopause, and BMI (results not shown).

Among both nulliparous and parous women, breast cancer risk increased with decreasing age at menarche. Breast cancer risk increased with increasing height among parous women, whereas the trend was less clear among nulliparous women.

For age at menopause, there was a strong positive association with breast cancer risk among parous women, but no clear trend among nulliparous women.

Using the entire follow-up period, there was a positive association of BMI with breast cancer risk in both strata of parity (results not shown). After stratification by age, BMI was not associated with breast cancer risk in the age group 55–69 years. However, for women 70 years and older, there was a clear positive association of BMI with breast cancer risk.

The HRs for age at menarche, height and BMI were homogenous across the two strata of parity, whereas the trend of increasing risk with increasing age at menopause was more pronounced among parous women.

The assessments of synergism between parity and age at menarche, age at natural menopause, height, and BMI are presented in Table 4. We found that the effects of age at menarche and height did not substantially differ for nulliparous and parous women. There was some indication that nulliparity and late age at menopause could decrease each other's effect, although the statistical power to detect such an antagonistic interaction was limited (API −0.24, 95% CI −0.55, 0.08). For the combined effects of nulliparity and high BMI, the results indicated independent effects in the age group 55–69 years, and synergistic effects after 70 years of age (API 0.21, 95% CI 0.04, 0.39).

Adjustments for potentially confounding factors had no material influence on the results. Among parous women, controlling for number of full-term pregnancies and age at first full-term pregnancy did not influence the associations of age at menarche, age at natural menopause, height, or BMI with breast cancer risk (results not shown).

## Discussion

In this prospective cohort study, we found that overweight may enhance the effect of nulliparity on breast cancer risk in elderly women. The results suggest that one out of five breast cancer cases among nulliparous and overweight women may be attributed to a synergistic effect of these factors.

Although there has been extensive research on the association of menstrual and anthropometric factors on breast cancer risk (Kelsey *et al*, 1993; Friedenreich, 2001), most studies have not shown results stratified by parity. In a meta-analysis of seven cohort studies, interaction between parity and anthropometric factors was assessed on a multiplicative scale, and showed no evidence for interaction in relation to breast cancer risk (Van den Brandt *et al*, 2000). Also, the results of a case–control study from Japan suggested no interaction on a multiplicative scale between parity and menstrual and anthropometric factors (Hirose *et al*, 2003). In contrast to our results, the Japanese investigators found no effect of age at menarche, but a strong positive association of age at menopause among nulliparous women. In a case–control study from Italy, the associations of menstrual factors with breast cancer risk within groups of parity were similar to the results of the Japanese study (La Veccia *et al*, 1992). Our results partly confirm these earlier observations, we found no evidence that the relative risks for breast cancer associated with age at menarche, height and BMI were different between parous and nulliparous women, as the tests for interaction were not statistically significant on a multiplicative scale. However, absence of association modification on a multiplicative scale does not exclude effect modification in a causal sense (Greenland *et al*, 2008). In the case of parity and BMI, our results suggest that the two factors do not act independently, but rather amplify each other's effect. On the other hand, parity and age at menopause showed a possible antagonistic interaction, both on the multiplicative and, more importantly, on the additive scale.

The interaction that we observed may advance our understanding of how different risk factors can act together. If confirmed by others, the finding might also be relevant for preventive strategies (Thompson, 1991), as our results suggest that maintaining a healthy body weight could potentially prevent a relatively higher proportion of breast cancer cases among nulliparous than among parous women.

A possible synergism between nulliparity and obesity suggests that there might be pathways where both factors are necessary for the development of breast cancer (Ahlbom and Alfredsson, 2005; Greenland *et al*, 2008). A high BMI is associated with higher production of oestrogen in adipose tissues, altered mammary stromal environment and higher insulin levels, and it has been suggested that these factors may mediate the positive association of BMI with breast cancer risk in postmenopausal women (Friedenreich, 2001; McCready *et al*, 2010). Results from a recent study suggested that insulin may mediate most of the increased risk associated with a high BMI (Gunter *et al*, 2009). Insulin is believed to increase breast cancer risk through mitogenic, and not mutagenic mechanisms, and may therefore act at a late stage in cancer development (Vineis *et al*, 2010). A synergism between BMI and nulliparity seems consistent with current models for breast cancer development (Siemiatycki and Thomas, 1981; Vineis *et al*, 2010), as nulliparous women may have a higher number of breast cells with a malignant potential (Britt *et al*, 2007), and overweight may promote cell growth (Vineis *et al*, 2010).

The positive association of BMI with breast cancer risk, and the synergism between BMI and nulliparity, were restricted to older postmenopausal women. A stronger association of BMI with breast cancer risk among older postmenopausal women has been described previously, and might be explained by a higher cumulative exposure due to longer duration of obesity, or that a certain induction period may be necessary (Yong *et al*, 1996; Galanis *et al*, 1998).

Late age at menopause is believed to increase breast cancer risk through a longer period of exposure to ovarian sex steroids (Kelsey *et al*, 1993). It has been suggested that nulliparous women may be more vulnerable to the carcinogenic effects of oestrogens (Russo and Russo, 2006), and therefore, the indication for a possible antagonism between a late age at menopause and nulliparity that we observed, was unexpected. The finding should also be interpreted with caution because of limited statistical power. In general, an antagonism between two factors may indicate competition between factors in pathways where only one of the factors is a necessary cause of the disease (Greenland *et al*, 2008).

The high attendance and prospective nature of our study minimise the possibility for bias in selection or information, although non-differential misclassification of the collected information would be expected and most likely result in conservative estimates of effect. Reporting of new cancers to the Cancer Registry of Norway is mandatory by law, and the data of the Cancer Registry are linked to the national Cause of Death Registry, which ensures a nearly complete registration of cancer cases (Lund, 1981). The women in this study are too old to have experienced any organised mammography screening programme (Kalager *et al*, 2009), and therefore, most if not all cancer cases, were clinically detected.

We had no information on family history of breast cancer, alcohol intake, physical activity, or use of exogenous hormones (oral contraceptives or hormone replacement therapy), all of which could have potentially confounded the results. However, given the birth year of the youngest women, the use of oral contraceptives in the cohort is likely to be negligible, and hormone replacement therapy may be relevant only for a small proportion of the youngest participants (Bergsjø, 1984). Although we adjusted for several potential confounders in the multivariable analyses, we cannot exclude the possibility of uncontrolled confounding. Nevertheless, any remaining confounder potentially able to influence our results considerably would need to (1) be strongly associated with breast cancer risk and the exposures that we studied, and (2) be unrelated to the potential confounders that were included in our models.

In the evaluation of synergism, we carried out five tests of interactions. Although these tests were dependant, caution is needed when interpreting our findings. In general, considerably higher statistical power is required to detect interactions than to detect single exposure–disease associations. Our study included a large number of elderly nulliparous women and an exceptionally long follow-up. Nevertheless, the statistical power to detect interactions was limited.

In conclusion, our findings suggest a possible synergistic effect of nulliparity and BMI in elderly women on breast cancer risk. If confirmed by others, this finding may advance our understanding of breast cancer, and might eventually lead to better preventive strategies.

## Figures and Tables

**Table 1:** Summary of the number of women eligible, the exclusions made and the number of women included in analyses

	**Exposure**		
	**Age at menarche**	**Age at natural menopause**	**Height and BMI**
Participants (*n*)	63 041	63 041	50 061
Eligible for analyses (*n*)^a^	62 108	62 108	49 624
			
*Exclusions (*n)			
Previous breast cancer diagnosis	759	759	651
Missing information^b^	3158	3158	2332
Exposure not applicable^c^		40 601	
Surgical menopause		451	
Unreliable measurement			1689
			
Included in analyses (*n*)	58 191	20 297	44 952

Abbreviation: BMI=body mass index.

a Women who could be followed from age 55 (excluding women who died or emigrated before age 55).

b Missing information on parity or covariates.

c Women who were premenopausal at the time of the interview.

**Table 2:** Characteristics at baseline of 58 191 women at risk of breast cancer, by parity

	**Nulliparous women (*n*=10 765)** a	**Parous women** (***n*=47 426)**^b^	**Total (*n*=58 191)**
Mean age in 1961 (s.d.)^c^	51.7 (11.0)	49.0 (10.8)	49.5 (10.9)
Mean age at height and weight measurement (s.d.)^d^	58.8 (10.4)	56.2 (9.9)	56.6 (10.0)
Mean age at menarche (s.d.)	14.3 (1.4)	14.2 (1.4)	14.2 (1.4)
Mean age at natural menopause (s.d.)	47.9 (4.6)	48.3 (4.2)	48.2 (4.3)
Mean height, cm (s.d.)	160.5 (6.0)	160.7 (5.6)	160.7 (5.7)
Mean body mass index, kg m^−2^ (s.d.)	25.4 (4.1)	26.1 (4.3)	26.0 (4.2)
			
*Marital status* (%)			
Never married	5681 (52.8)	548 (1.2)	6229 (10.7)
Ever married	5084 (47.2)	46 878 (98.8)	51 962 (89.3)
			
*County* (%)			
Nord-Trøndelag	3237 (30.0)	16 106 (34.0)	19 343 (33.2)
Aust-Agder	2816 (26.2)	9993 (21.1)	12 809 (22.0)
Vestfold	4712 (43.8)	21 327 (45.0)	26 039 (44.7)
			
*Community of residence* (%)			
Urban	2839 (26.4)	9535 (20.1)	12 374 (21.3)
Rural	7926 (73.6)	37 891 (79.9)	45 817 (78.7)
			
*Occupation (own or husband's,* %)			
Professional, private enterprise	3530 (32.8)	12 634 (26.6)	16 164 (27.8)
Manual	2890 (26.8)	22 048 (46.5)	24 938 (42.9)
Domestic and other work	4345 (40.4)	12 744 (26.9)	17 089 (29.4)

a Women with no full-term pregnancy.

b Women with one or more full-term pregnancies.

c Age in 1961 is not equivalent to age at start follow-up for all participants.

d Age at height and weight measurement is not equivalent to age at start follow-up for all participants.

**Table 3:** Risk factors for breast cancer by parity, in postmenopausal women from three counties in Norway

	**Nulliparous women** a				**Parous women** b				
	**No. of person-years**	**No. of cases**	**Rate** c	**Hazard ratio (95% confidence interval)**	**No. of person-years**	**No. of cases**	**Rate**	**Hazard ratio (95% confidence interval)**	**P_LR test_** d
Parity	248 382	636	256	1.26 (1.12, 1.42)	1152 054	2254	196	1 (reference)	
									
*Age at menarche, years* e									
⩽13	68 436	202	295	1.24 (1.02, 1.64)	337 112	731	217	1.12 (1.01, 1.24)	
14	83 659	201	240	1 (reference)	374 377	732	196	1 (reference)	
15	54 746	142	259	1.08 (0.87, 1.34)	258 930	475	183	0.94 (0.83, 1.05)	
⩾16	41 541	91	219	0.92 (0.72, 1.18)	181 635	316	174	0.89 (0.78, 1.02)	
P_*trend*_^f^				0.04				<0.001	0.88
									
*Height, cm* g									
<155	21 718	56	258	0.90 (0.65, 1.23)	98 269	167	170	0.73 (0.61, 0.86)	
155–159	40 665	111	273	0.99 (0.77, 1.27)	226 554	436	192	0.85 (0.75, 0.96)	
160–164	52 839	142	269	1 (reference)	291 489	642	220	1 (reference)	
165–169	30 739	87	283	1.09 (0.83, 1.42)	171 802	359	209	0.98 (0.86, 1.11)	
⩾170	13 004	36	277	1.11 (0.77, 1.61)	53 799	137	255	1.22 (1.01, 1.47)	
P_*trend*_				0.24				<0.0001	0.23
									
*Age at menopause, years* g									
<45	17 557	37	211	0.85 (0.57, 1.26)	51 452	63	122	0.62 (0.47, 0.84)	
45–49	38 682	102	264	1.02 (0.77, 1.36)	122 937	192	156	0.78 (0.64, 0.94)	
50–54	39 408	104	264	1 (reference)	131 848	272	206	1 (reference)	
⩾55	2548	7	275	1.01 (0.47, 2.17)	10 560	31	294	1.34 (0.93, 1.96)	
P_*trend*_				0.55				<0.0001	0.04
									
**Breast cancer risk among women followed from age 55 to 69 years**									
*Body mass index, kg m* ^−*2*g^									
<25	32 180	64	199	1 (reference)	161 590	233	144	1 (reference)	
25–29	24 434	43	176	0.88 (0.59, 1.30)	149 325	240	161	1.13 (0.94, 1.36)	
⩾30	8638	19	220	1.09 (0.64, 1.85)	65 233	108	166	1.17 (0.93, 1.49)	
P_*trend*_				0.99				0.14	0.60
									
**Breast cancer risk among women followed from age 70 years or older**									
*Body mass index, kg m* ^−*2*g^									
<25	43 019	106	246	1 (reference)	180 234	384	213	1 (reference)	
25–29	37 388	139	372	1.50 (1.16, 1.94)	199 634	494	247	1.17 (1.02, 1.34)	
⩾30	13 619	61	448	1.76 (1.27, 2.44)	87 601	287	328	1.56 (1.33, 1.83)	
P_*trend*_				<0.001				<0.0001	0.26

a Women with no full-term pregnancy.

b Women with one or more full-term pregnancies.

c Incidence per 100 000 person-years.

d *P*-value from likelihood ratio test (comparison of model with and without interaction term).

e Adjusted for age, birth cohort, county of residence, urban or rural community of residence, marital status, occupation (own or husband's).

f *P*-value for trend across exposure categories.

g Adjusted for age, birth cohort, county of residence, urban or rural community of residence, marital status, occupation (own or husband's) and age at menarche.

**Table 4:** Assessment of synergism between parity and other breast cancer risk factors among postmenopausal women from three counties in Norway

	**No. of person-years**	**No. of cases**	**Rate** a	**Hazard ratio (95% CI)**
*Age at menarche and parity* b				
Late menarche (⩾15 years) and parous^c^	440 565	791	180	1 (reference)
Early menarche (<15 years) and parous	711 489	1463	206	1.15 (1.05, 1.25)
Late menarche (⩾15 years) and nulliparous^d^	96 286	233	242	1.30 (1.10, 1.54)
Early menarche (<15 years) and nulliparous	152 096	403	265	1.42 (1.23, 1.64)
API (95% CI)^e^				−0.02 (−0.18, 0.15)
				
*Height and parity* f				
Short (<161 cm) and parous	387 264	751	194	1 (reference)
Tall (⩾161 cm) and parous	454 648	990	218	1.18 (1.07, 1.30)
Short (<161 cm) and nulliparous	74 511	195	262	1.33 (1.11, 1.59)
Tall (⩾161 cm) and nulliparous	84 454	237	281	1.50 (1.27, 1.78)
API (95% CI)				−0.01 (−0.20, 0.19)
				
*Age at menopause and parity* f				
Early menopause (<50 years) and parous	174 389	255	146	1 (reference)
Late menopause (⩾50 years) and parous	142 408	303	213	1.39 (1.17, 1.66)
Early menopause (<50 years) and nulliparous	56 239	139	247	1.55 (1.21, 1.98)
Late menopause (⩾50 years) and nulliparous	41 957	111	265	1.57 (1.20, 2,05)
API (95% CI)				−0.24 (−0.55, 0.08)
				
**Breast cancer risk among women followed from age 55 to 69 years**				
*BMI and parity* f				
Normal BMI (<26 kg m^−2^) and parous	200 041	293	146	1 (reference)
High BMI (⩾26 kg m^−2^) and parous	176 106	288	164	1.13 (0.96, 1.34)
Normal BMI (<26 kg m^−2^) and nulliparous	39 062	73	187	1.17 (0.86, 1.59)
High BMI (⩾26 kg m^−2^) and nulliparous	26 190	53	202	1.29 (0.92, 1.80)
API (95% CI)				−0.01 (−0.38, 0.36)
				
**Breast cancer risk among women followed from age 70 years or older**				
*BMI and parity* f				
Normal BMI (<26 kg m^−2^) and parous	228 591	507	222	1 (reference)
High BMI (⩾26 kg m^−2^) and parous	238 878	658	275	1.23 (1.09, 1.39)
Normal BMI (<26 kg m^−2^) and nulliparous	53 035	141	266	1.25 (1.00, 1.55)
High BMI (⩾26 kg m^−2^) and nulliparous	40 992	165	403	1.88 (1.53, 2.30)
API (95% CI)				0.21 (0.04, 0.39)

Abbreviations: API=attributable proportion due to interaction; BMI=body mass index, CI=confidence interval.

a Incidence per 100 000 person-years.

b Adjusted for age, birth cohort, county of residence, urban or rural community of residence, marital status, occupation (own or husbands).

c Parous women defined as women with one or more full-term pregnancies.

d Nulliparous women defined as women with no full-term pregnancy.

e Attributable proportion due to interaction.

f Adjusted for age, county of residence, urban or rural community of residence, marital status, occupation (own or husbands) and age at menarche.
